# Patient safety skills in primary care: a national survey of GP educators

**DOI:** 10.1186/s12875-014-0206-5

**Published:** 2014-12-17

**Authors:** Maria Ahmed, Sonal Arora, John McKay, Susannah Long, Charles Vincent, Moya Kelly, Nick Sevdalis, Paul Bowie

**Affiliations:** Centre for Primary Care, Faculty of Medical and Human Sciences, The University of Manchester, Oxford Road, Manchester, M13 9PL UK; Department of Surgery and Cancer, Imperial College London, St Mary’s Hospital, Praed Street, London, W2 1NY UK; NHS Education for Scotland, 2 Central Quay, Glasgow, G3 8BW UK; St Mary’s Hospital, Imperial College Healthcare NHS Trust, Praed Street, London, W2 1NY UK; Department of Experimental Psychology, University of Oxford, Tinbergen Building, 9 South Parks Road, Oxford, OX1 3UD UK; Wright Fleming Building, Department of Surgery and Cancer, Imperial College London, Norfolk Place, London, W2 1PG UK; Institute of Health and Wellbeing, University of Glasgow, Glasgow, UK

**Keywords:** General practice, Patient safety, Medical education, Skills

## Abstract

**Background:**

Clinicians have a vital role in promoting patient safety that goes beyond their technical competence. The qualities and attributes of the safe hospital doctor have been explored but similar work within primary care is lacking. Exploring the skills and attributes of a safe GP may help to inform the development of training programmes to promote patient safety within primary care.

This study aimed to determine the views of General Practice Educational Supervisors (GPES) regarding the qualities and attributes of a safe General Practitioner (GP) and the perceived trainability of these ‘safety skills’ and to compare selected results with those generated by a previous study of hospital doctors.

**Methods:**

This was a two-stage study comprising content validation of a safety skills questionnaire (originally developed for hospital doctors) (Stage 1) and a prospective survey of all GPES in Scotland (n = 691) (Stage 2).

**Results:**

Stage 1: The content-validated questionnaire comprised 66 safety skills/attributes across 17 broad categories with an overall content validation index of 0.92.

Stage 2: 348 (50%) GPES completed the survey. GPES felt the skills/attributes most important to being a safe GP were honesty (93%), technical clinical skills (89%) and conscientiousness (89%). That deemed least important/relevant to being a safe GP was leadership (36%). This contrasts sharply with the views of hospital doctors in the previous study. GPES felt the most trainable safety skills/attributes were technical skills (93%), situation awareness (75%) and anticipation/preparedness (71%). The least trainable were honesty (35%), humility (33%) and patient awareness/empathy (30%). Additional safety skills identified as relevant to primary care included patient advocacy, negotiation skills, accountability/ownership and clinical intuition (‘listening to that worrying little inner voice’).

**Conclusions:**

GPES believe a broad range of skills and attributes contribute to being a safe GP. Important but subtle differences exist between what primary care and secondary care doctors perceive as core safety attributes. Educationalists, GPs and patient safety experts should collaborate to develop and implement training in these skills to ensure that current and future GPs possess the necessary competencies to engage and lead in safety improvement efforts.

**Electronic supplementary material:**

The online version of this article (doi:10.1186/s12875-014-0206-5) contains supplementary material, which is available to authorized users.

## Background

Historically, patient safety improvement efforts have focussed on the secondary care (hospital) setting. This was driven in part due to a historical focus of early research [1] as well as policy-makers/implementers (e.g. the former National Patient Safety Agency in the UK) on hospital-delivered care. The in-hospital focus was also due to high-profile failings in healthcare within this sector, exemplified most recently by the case of Mid-Staffordshire NHS Trust in the UK [2,3]. However, in countries with strong primary health care systems such as the UK, Canada and the Netherlands, the majority of patient consultations occur within the primary care (community) setting [4]. In the UK, for example, over 300 million consultations take place annually in primary care [5]. Recent evidence suggests that between 1-2% of such consultations may involve an adverse event with most involving polypharmacy, multiple complex conditions and systems issues such as inadequate communication and information sharing processes [6]. While many of these events will be minor, there is always potential for serious harm [6]. Primary care differs from other healthcare sectors in the volume of undifferentiated clinical presentations and the need to manage the associated uncertainty [7]. The importance of patient safety in primary care is therefore increasing in recognition, including commitments of research funding [8] and at health policy level [9].

A wide range of factors spanning individual, team and systems influences are known to contribute to adverse events in healthcare [10]. In recent years, there has been growing attention on the role of frontline clinicians in promoting patient safety [11]. It is not sufficient for clinicians to be technically competent in their practice. A wide range of ‘non-technical skills’ including communication, team-working, situation awareness, decision-making and problem-solving are now well-evidenced aspects of delivering high-quality care [12]. Thinking more broadly, the qualities and attributes of the ‘safe practitioner’ have been previously explored [13]. However, these efforts have focussed on clinicians within secondary care and particularly the craft specialties of surgery and anaesthesia [14,15]. As frontline clinicians in primary care, General Practitioners (GPs; termed ‘community physicians’ in the US) have a crucial role in promoting patient safety. It is likely that the complex and inherently uncertain environment of primary care requires a different set of ‘safety skills’, particularly given the ageing patient population and poly-pharmacy that GPs manage on a daily basis.

Education and training is recognised as an essential means of equipping clinicians with the requisite ‘safety skills’ to improve patient safety in the workplace [16]. Indeed a recent survey of primary care physicians and researchers considered education and training as one of the most important strategies to improve patient safety in primary care [4]. However, the provision of explicit training in patent safety within primary care settings is lacking [4], for example training in significant event analysis, human factors and quality improvement methods. The latest iteration of the UK Royal College of General Practitioners (RCGP) curriculum includes a comprehensive statement on patient safety and quality of care [17]. However, despite this, the delivery of safety training in practice is poor, as evidenced in a recent international study across countries with a strong primary care system [4]. Moreover, the attitudes towards and engagement in key risk management interventions such as significant event analysis varies widely amongst GPs [18,19] and the wider primary care team [20].

Exploring the skills and attributes of a safe GP may help to inform the development of training programmes to support GPs and their teams in ensuring and promoting a positive safety culture in the workplace. As a first step to this, and building on similar research with hospital doctors [13], the main objective of this study was to determine the views of GP Educational Supervisors (GPES) regarding the qualities and attributes of a safe GP and the perceived trainability of these ‘safety skills’. Our secondary objective was to compare selected results with those generated by the previous study with hospital doctors [13].

## Methods

This was a two-stage study comprising content validation of a safety skills questionnaire for use within primary care [13] and a prospective cross sectional survey of GP Educational Supervisors (GPES). As in the original hospital study, our objective was to explore the views of frontline clinicians – in this case GPs. Specifically, we chose GP educational supervisors as participants for this study as in addition to their clinical duties they have a structured role in the education and training of GPs of which a significant proportion includes teaching clinical and systems based approaches to safety management, and so are ‘informed’ on these issues. Additionally all GPES have a minimum of five years in frontline general medical practice and have undergone a structured, modular training programme that requires the GP to demonstrate via video consultation that they are safe practitioners and are able to satisfactorily apply the significant event analysis technique [21]. This makes them of interest to the study because it differentiates them from the rest of the frontline GP population, as well as GP academics and researchers with an interest in patient safety.

### Setting

General Practice specialty training (GPST) across the four postgraduate medical deaneries in Scotland is 3 to 4 years in length. Of this, 18 months are spent in a GP setting with the remainder of the time in a variety of hospital posts. Each trainee has a named GPES throughout the length of their training programme. The curriculum for GPST is provided by the RCGP [17] and must be covered during training and evidenced within the trainees’ e-portfolio. Patient safety and quality of care are part of the curriculum but there are no defined methods of teaching or assessing this important area, thereby introducing significant potential for variation in educational provision, with possible implications for patient care.

### Stage one: content validation of safety skills questionnaire for primary care

A previously published questionnaire of safety skills formed the basis of this study [13]. This original questionnaire was developed via online survey and a subsequent face-to-face focus group of ten experienced patient safety researchers (backgrounds in medicine, surgery and psychology) to elicit their views of key safety skills. In order to avoid altering the underlying constructs in any way, the 73 safety skills proposed by participants were used verbatim to form items for the questionnaire, which sought responses regarding perceived importance and trainability of each item on a 5-point Likert scale.

For this study, the questionnaire was initially reviewed and content validated during a 90-minute medical education workshop involving all 24 trained GPES from a single specialty training group in the west of Scotland. This phase was important to ensure that the subsequent questionnaire used for stage two contained the appropriate safety skills that would be relevant to GPs - particularly as the previous iteration was intended for use by hospital doctors. All participants were provided with the original questionnaire of 73 items and asked to independently rate the relevance of each item to being a safe GP. A four-point Likert scale [22] was used to indicate level of relevance (1 = not relevant; 2 = unable to assess relevance without item revision or item in need of such revision that it would no longer be relevant; 3 = relevant but needs minor alteration; 4 = very relevant and succinct). Free-text comments against each item were also encouraged from participants.

Data were analysed using SPSS v19.0. For each item, a Content Validity Index (CVI) was calculated to assess the level of agreement between the 24 GPES, based on the proportion with a rating of 3 or higher (on the 4-point scale). The CVI is a method to quantitatively summarise expert views, expressed on a numerical scale, on a topic – in our case on the elements that make up a safe GP. A cut-off of 0.80 is typically set in research contexts, such that items that score >0.80 across a pool of experts are considered to have reached agreement (i.e. on their importance, or relevance) and hence they are maintained, whereas those that fail to do so are discarded, or amended [22]. Using this criterion, items with a CVI < 0.80 were removed from the questionnaire as these were perceived by the experienced GPES to be of less relevance to being a safe GP [23]. Two independent researchers also conducted a qualitative analysis of free-text comments. Items that had a high CVI > 0.80 and received strong support through the qualitative analysis were reviewed by a multi-disciplinary panel consisting of the original questionnaire developers, patient safety experts, GPES and senior educationalists to form the final content-validated questionnaire to be used in stage two.

### Stage two: survey of GP educational supervisors

All 691 GPES across Scotland were identified from the NHS Education for Scotland organisational databases and invited to participate in the study. The modified questionnaire from stage one was administered electronically using ‘QuestBack’ software (www.questback.co.uk). For each listed skill/attribute, participants were asked to indicate (i) its perceived importance/relevance to primary care and (ii) its perceived trainability. Both questions were answered on 5-point Likert scales. Participants were also invited to suggest additional skills/attributes not listed within the questionnaire. Questionnaire responses were anonymous and participation was voluntary, with subjects free to exit the survey at any point. Two-weekly reminders were sent between November 2011 and February 2012 to non-respondents. Ethical approval was not required for this study.

Descriptive statistics were calculated using SPSS v 19.0 (proportion agreeing (score = 4 or 5), disagreeing (score = 1 or 2) and neither agreeing/disagreeing (score = 3) for each item). For the purposes of data analysis the importance and trainability data for each questionnaire item (i.e., skill) were grouped into 17 broad categories (of the original questionnaire developed with hospital doctors). As per the secondary objective of this study, this enabled comparisons with the original hospital study. This latter data-set comprised questionnaire responses from 50 physicians and surgeons. Additional items suggested by respondents were analysed for emerging themes by two independent clinical reviewers.

## Results

### Stage one: content validation of safety skills questionnaire for primary care

**Figure 1:**
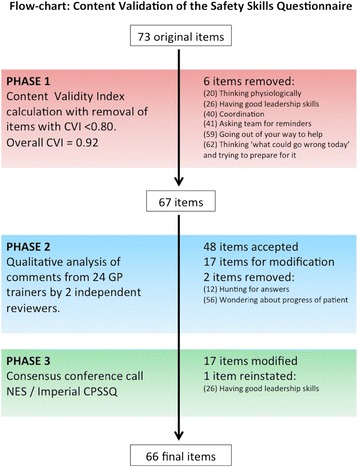
**Content validation of the Safety Skills Questionnaire for GPESs.**

The content validation process is summarised in Figure 1. Initial content validation of the original 73-item safety skills questionnaire resulted in 6 items being removed (CVI < 0.80). Qualitative analysis of respondents’ comments by two researchers resulted in 48 items being included with no modifications; 17 items being modified via multidisciplinary consensus, and 2 items being removed. One item that had been initially removed (‘*Having good leadership skills*’) was reinstated as the multidisciplinary panel felt it would be important to explore the views of the broader GPES community given national interest in medical leadership and management [24]. The final content validated questionnaire for safety skills in primary care therefore comprised 66 skills/attributes grouped into 17 broad categories (Additional file 1).

### Stage two: survey of GP educational supervisors

A total of 348 GPES completed the survey (response rate = 50%) between November 2011 and February 2012, with all respondents indicating levels of agreement with all statements to provide a full data set.

#### Importance/relevance of safety skills

**Figure 2:**
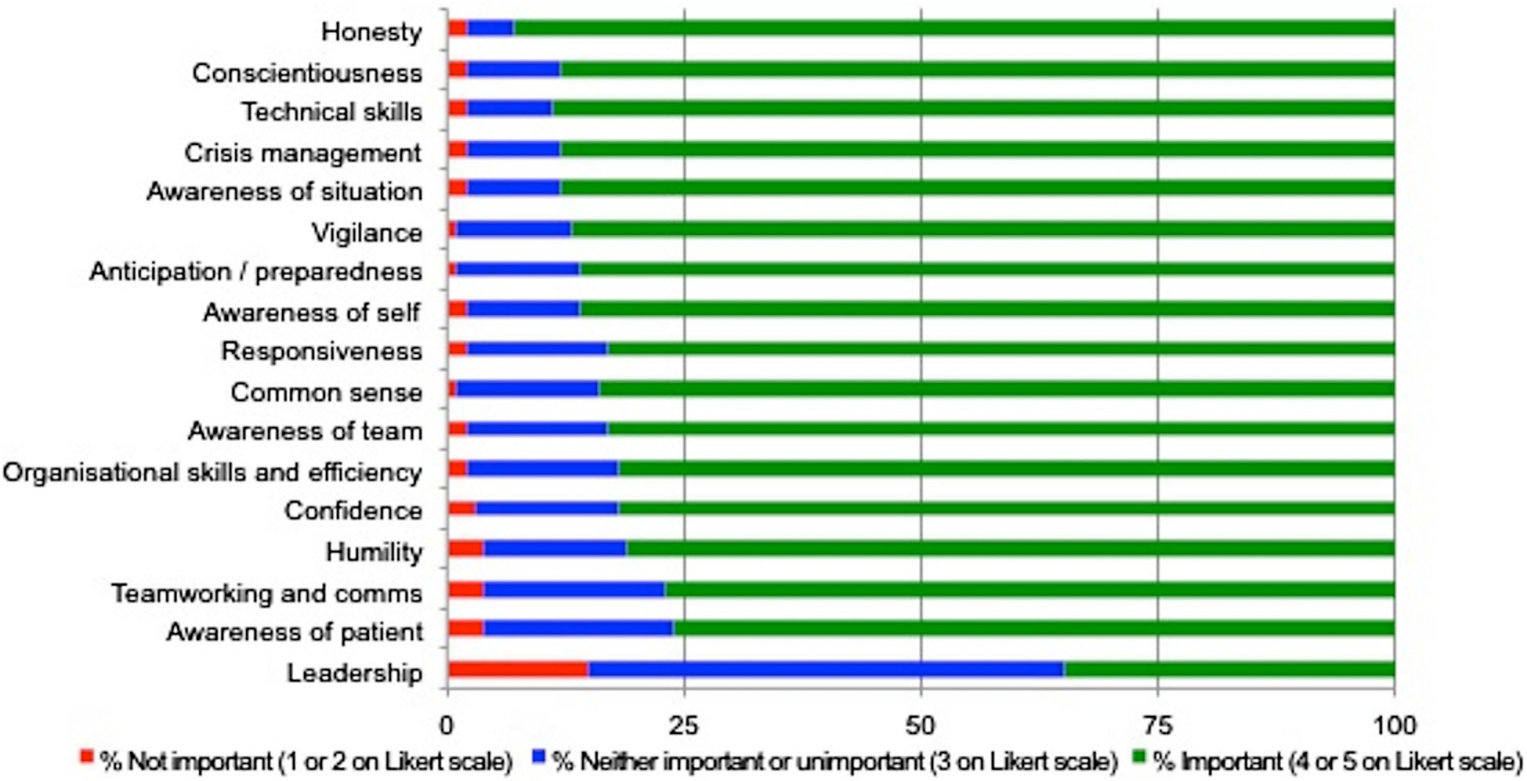
**Perceived importance of safety skills.**

Respondents felt that the majority of skills were important to being a safe GP (score = 4 or 5). The skills/attributes deemed most important to being a safe GP were honesty (93%), technical/clinical skills (89%) and conscientiousness (89%). That deemed least important/relevant was leadership (36%) Figure 2.

#### Trainability of safety skills

**Figure 3:**
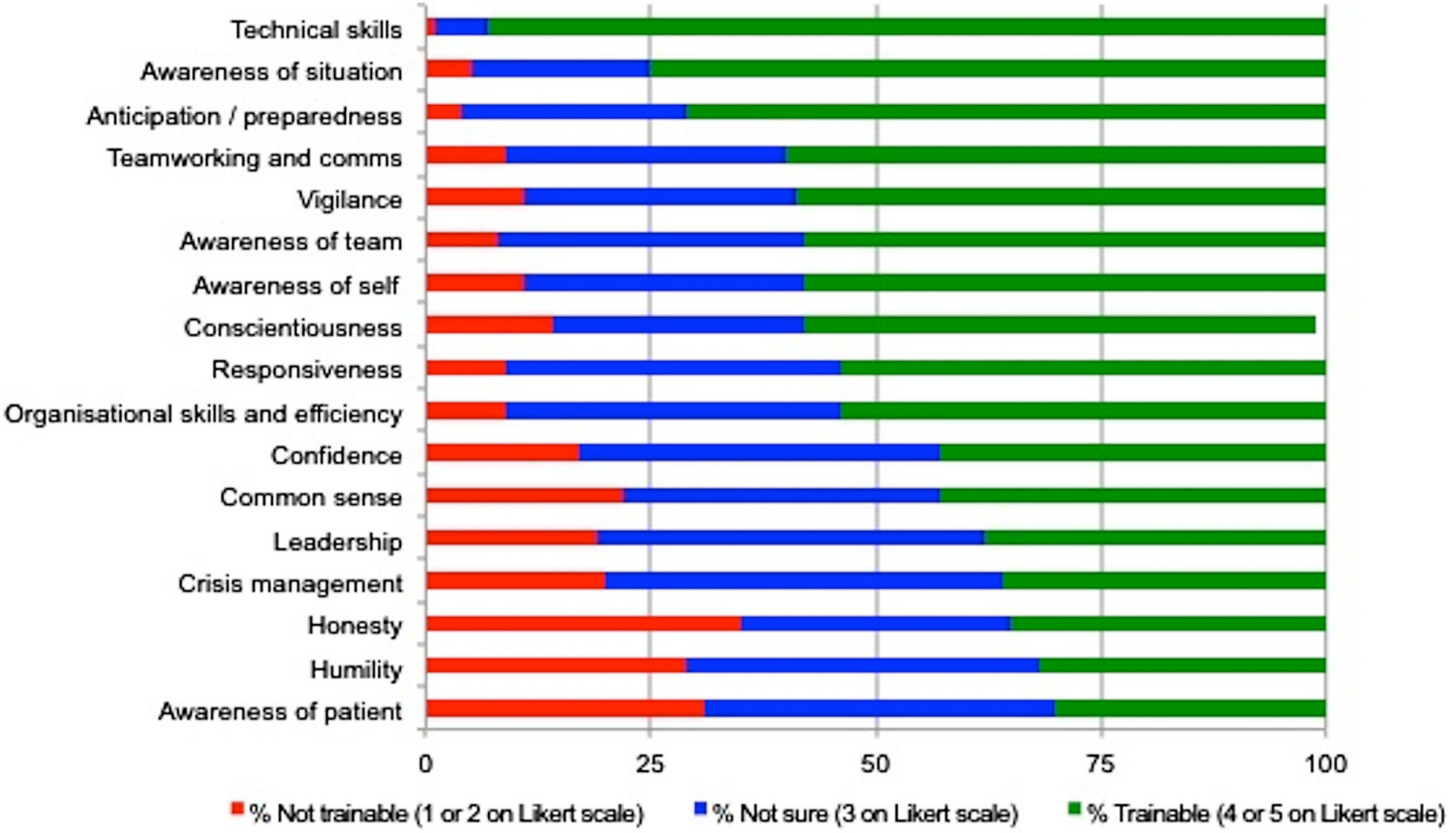
**Perceived trainability of safety skills.**

There was lower agreement regarding the perceived trainability of the safety skills/attributes. GPES felt the most trainable safety skills/attributes were technical/clinical skills (93%), situation awareness (75%) and anticipation/preparedness (71%). The least trainable were honesty (35%), humility (33%) and patient awareness/empathy (30%) Figure 3.

#### Additional safety skills proposed

Respondents proposed an additional 169 safety skills/attributes as being important as a GP. The majority of these matched closely with items already listed within the questionnaire. However, there were a number of novel skills/attributes proposed by respondents, which are displayed in Table 1.

**Table 1:** **Novel ‘safety skills’ proposed by GPES respondents**

**Category**	**Safety skill/attribute**	**Illustrative quote**
***Novel ‘safety skills’ falling under existing categories***		
Awareness of patient	Patient focussed	*‘Patient-centredness’*
	Patient advocacy	*‘Ability to be the patient’s advocate’*
Awareness of self	Having an appropriate work-life balance	*‘Engages in social life and has external hobbies’*
		*‘Recognising personal burnout / when you no longer care about your job’*
Teamwork and communication	Negotiation skills	*‘Negotiating with team members regarding division of workload’*
	Conflict resolution skills	*‘Dealing with conflict’*
	Debriefing skills	*‘Debriefing or time-out after difficult encounters’*
	Ensuring clear documentation	*‘Full and sufficiently detailed clinical consultation record keeping’*
***Novel ‘safety skills’ not falling under existing categories***		
	Taking ownership/accountability	*‘Accepting ownership/responsibility’*
		*‘Sense of personal responsibility for effective patient care’*
		*‘Continuity of care’*
	Intuition	*‘Listening to your sixth sense (i.e. recognising your own instincts)’*
		*‘Listening to that worrying little ‘inner voice’…’*

### Comparison with hospital study findings

There was generally good agreement between what GPESs in this study, and hospital doctors of the recent study [13] perceive as core qualities/attributes of a safe practitioner. As in this study, technical skills and honesty were perceived to be two of the most important skills/attributes of a safe doctor in the hospital study (for both items, 98% respondents agreed or strongly agreed). ‘Awareness of patient’ (includes empathy) was ranked relatively low in importance in both studies (78% respondents agreed/strongly agreed in this study, 82% in the hospital study). In contrast, whilst leadership was perceived to be important by only 36% of respondents in this study, 93% of doctors in the hospital study believed it to be important to being a safe doctor.

Regarding skill trainability, technical/clinical skills were considered the most trainable in both studies. Skills relating to team-working, preparedness, vigilance and situational awareness also featured highly in perceived trainability across both studies, which are all well-recognised non-technical skills [12]. In contrast, ‘softer’ skills and attributes such as confidence, humility and common sense were ranked relatively lower in both studies.

## Discussion

### Summary

GPESs in this study recognised a broad range of skills/attributes associated with being a safe GP. Importantly, many of these were judged to be trainable. Not surprisingly, technical/clinical skills were considered important and the most trainable. Least trainable were attributes that would constitute professional values; yet these are crucial to the effective performance of any clinical practitioner, the learning culture in the practice and therefore patient safety [17,25]. Interestingly, leadership was considered the least important skill/attribute to being a safe GP, which contrasts sharply with the views of hospital doctors [13]. This may reflect a difference in the way that GPs understand and interpret the concept of leadership in respect of safety, as compared to hospital doctors. For GPs, safety may reside much more in individual practice and care, rather than in the leadership of a team and they may conflate ‘leadership’ with ‘management’. In contrast, hospital doctors often have more clearly defined leadership roles as part of clinical teams at the frontline and also as part of clinical directorates at managerial level. This may make leadership in respect to safety a more recognised construct within secondary care.

The additional novel skills/attributes proposed by respondents further suggest that there are important, albeit subtle differences in the perceived necessary safety skill-set between primary and secondary care doctors. For example, the fundamental feature of general practice in coordinating the effective provision of holistic, long-term patient care may be reflected in the additional item suggested ‘taking ownership/accountability’. This is in contrast to care in the hospital sector where the majority of patient contacts and follow-up are on a relatively short-term basis, with patients ultimately discharged back to the care of their GP. In a similar vein, that GPs are often the patients’ first port of call for medical advice (and the onus that this places on GPs) may be reflected in the additional item suggested ‘intuition – listening to that worrying little inner voice’. Interestingly, ‘awareness of patient’ was ranked relatively low by GPESs in both importance and perceived trainability. It is possible that this item is not fully understood. GPES and trainees are focused on ‘patient-centred’ consulting and determining the patient’s agenda, which is what is required to pass the RCGP membership examination. The proposed alternative terms ‘patient-focused’ and ‘patient advocacy’ reflect this specific context. In a similar vein, ‘negotiation skills’ (as a subset of teamwork and communication) was suggested as an additional item, perhaps emphasising this critical GP skill in negotiating a management plan with patients (as their advocates), and with other healthcare professionals, as ‘gatekeepers’ to secondary care.

### Comparison with existing literature

To the best of our knowledge, this is the first national survey to explore GPES’ views regarding the key skills and attributes necessary to being a safe GP. The views of GPs regarding what constitutes ‘patient safety’ have been previously explored, but the specific individual skills and attributes necessary to be a safe GP were largely overlooked when compared to team and systems level priorities [26]. Most of our study findings compare favourably with existing competency frameworks [17], but provide further insight into the potential prioritisation of competencies and what is trainable, as perceived by GPES from a patient safety perspective. For example, training in some safety-critical skills such as team-working and communication is widely available and researched. However, perhaps an added dimension is required to emphasise how these skill-sets interact and impact on the safety of the immediate workplace and the wider NHS. From this perspective there is now growing interest in educating the NHS workforce in human factors science [27]. A possible educational gap, therefore, is the need to move beyond a limited focus on the importance of ‘non-technical skills’ to patient safety to an appreciation of a more holistic understanding of the role and consequences of human-system interactions in the clinical workplace. Developing educational interventions for patient safety that focus on raising awareness of the workplace as a complex socio-technical system will be necessary [28].

### Strengths and limitations

The survey response rate of 50% was moderate, but comparable to other internet-based surveys in healthcare [29]. The large number of respondents was sufficient for analysis and inference. We did not capture demographic data in an attempt to reduce the questionnaire completion time and because we judged that, although important, this information was not particularly critical to the study purpose and interpretation of findings. We were therefore unable to compare the characteristics of responders and non-responders so the possibility of response bias cannot be excluded. Our findings may not therefore be generalisable from a GP educator perspective, or representative of the non-training GP community. Moreover, we acknowledge that this particular group of GPs may not be considered ‘experts’ and therefore the possibility of ‘unconscious incompetence’ exists, with GPES having differing interpretations of safety and a potential lack of knowledge/insight of safety systems and their role in individual errors when compared to patient safety ‘experts’. Related to this point is the technical limitation that using the CVI as a numerical cut-off for survey items may have resulted in items being discarded based on respondent consensus, whereas in fact safety experts would have scored them differently and potentially retained them. We acknowledge that there is inherent subjectivity in methods that rely on human judgement. Indeed, the additional items suggested by respondents may indicate a gap in the question set and/or interpretational issues on the part of the participants. Nonetheless we believe this study adds to our knowledge and understanding of this under-researched topic, with GPES well-placed to identify the safety attributes of a safe GP. All GPES must be deemed safe consulters prior to being accepted as educational supervisors, and patient safety competences both at individual (through medical appraisal) and system (practice) level are assessed at re-accreditation visits. Moreover, the GPES are all trained to assess and make judgements on trainees’ ability to practice (including safely) and can be called to account for these judgements.

### Implications for research and practice

These findings provide a basic educational platform on how to go about improving patient safety in primary care through targeted training of GPs in those safety skills/attributes which were perceived as a higher priority and judged to be trainable by experienced and informed frontline educators and clinicians. Importantly, this can help develop educational programmes in priority safety skills at different levels of training and career progression for GPs. Additionally, we may be able to agree a skill-set on which to base the development and alignment of assessment tools for testing the safety competencies of GPs, particularly those in training. Given increasing calls for primary care clinicians to engage in and lead in safety improvement efforts [30] our findings suggest a need to encourage GPs into more visible leadership positions and also emphasise how GPs are leaders in their own practices, although many may not explicitly recognise this. This is particularly prudent given the renewed moves towards primary care-led commissioning in England [31] and health and social care integration in Scotland [32]. Finally, this study also gives weight to the argument for extending GP specialty training to enable the acquisition of skills in patient safety, leadership and quality improvement in parallel to core clinical competencies - necessary for delivering safe, effective care for the 21^st^ century [17]. Future research should further explore GPs’ views on the concept of leadership as a safety skill in primary care. Repeating this study to explore the views of GP trainees and the wider primary care team could also provide invaluable insight into potential differences in views and may help to shape targeted training interventions for the whole primary care team in the future.

## Conclusion

The experienced GP educators in this study believe a broad range of skills and attributes are required to make a safe GP, although many are not explicitly recognised as core safety skills in specialty training or continuing professional development. Important but subtle differences exist between what primary care and secondary care doctors perceive as core safety attributes. Educationalists should work with GPs and patient safety experts to develop interventions aimed at developing these key safety skills/attributes. As we begin to understand the burden of avoidable harm in primary care better, such safety skills training will ensure that current and future GPs possess the necessary competencies to engage in efforts to enhance the safety of healthcare.

## Additional file

Additional file 1:
**Content validated Safety Skills Questionnaire for Primary Care.**

## Abbreviations

CVI: Content validation index
GP: General Practitioner
GPES: General Practitioner Educational Supervisor
GPST: General Practice specialty training
NHS: National Health Service
