# Supplementary material for: Patient safety skills in primary care: a national survey of GP educators
Source: BMC Fam Pract. 2014 Dec 17;15:206. doi: 10.1186/s12875-014-0206-5 (PMC4275946; doi:10.1186/s12875-014-0206-5)
Supplement: Additional file 1: — Content validated Safety Skills Questionnaire for Primary Care. [file 12875_2014_206_MOESM1_ESM.pdf]

SAFETY SKILLS SURVEY

Safety Skills are attributes that competent clinicians possess and that allow safe delivery of care. We are trying to understand what components make up these ***Safety Skills***.

For each of the skills/attributes listed below, please rate on a scale of 1 (least) to 5 (most):

a. How important and relevant is this skill/ attribute to being a safe GP?
b. How trainable is this skill/ attribute?

SKILL	Importance / Relevance to GP<br>Least Most	Trainable<br>Least Most
**1. Error awareness – recognising error-prone situations**	1 2 3 4 5	1 2 3 4 5
**2. Self awareness – recognising one's own mental and physical limitations**	1 2 3 4 5	1 2 3 4 5
**3. Awareness of the role of the team and its contribution**	1 2 3 4 5	1 2 3 4 5
**4. Reflective thinking**	1 2 3 4 5	1 2 3 4 5
**5. The ability to think clearly in a crisis**	1 2 3 4 5	1 2 3 4 5
**6. Disease pattern recognition & vigilance for deviation from patterns**	1 2 3 4 5	1 2 3 4 5
**7. Thinking & problem solving**	1 2 3 4 5	1 2 3 4 5
**8. Anticipation of organisational problems**	1 2 3 4 5	1 2 3 4 5
**9. Honesty**	1 2 3 4 5	1 2 3 4 5
**10. Taking feedback constructively**	1 2 3 4 5	1 2 3 4 5
**11. Decision-making**	1 2 3 4 5	1 2 3 4 5
**12. Common sense approach**	1 2 3 4 5	1 2 3 4 5

**13. Anticipation of deteriorating patient**	1 2 3 4 5	1 2 3 4 5
**14. Being non-judgemental**	1 2 3 4 5	1 2 3 4 5
**15. Humility**	1 2 3 4 5	1 2 3 4 5
**16. Having up to date knowledge/training**	1 2 3 4 5	1 2 3 4 5
**17. Taking an appropriate history and examination**	1 2 3 4 5	1 2 3 4 5
**18. Having good technical skills**	1 2 3 4 5	1 2 3 4 5
**19. Clear and effective communication**	1 2 3 4 5	1 2 3 4 5
**20. Know who, when, how to call for help appropriately**	1 2 3 4 5	1 2 3 4 5
**21. Ability to work well in a team**	1 2 3 4 5	1 2 3 4 5
**22. Continuous questioning of self and others**	1 2 3 4 5	1 2 3 4 5
**23. Having an appropriate level of confidence (not over- or under-confident)**	1 2 3 4 5	1 2 3 4 5
**24. Having good leadership skills**	1 2 3 4 5	1 2 3 4 5
**25. Responding to change in circumstances**	1 2 3 4 5	1 2 3 4 5
**26. Being able to speak up about error**	1 2 3 4 5	1 2 3 4 5
**27. Being open about error**	1 2 3 4 5	1 2 3 4 5
**28. Not letting one's own personal issues interfere with patient care**	1 2 3 4 5	1 2 3 4 5
**29. Being courteous and considerate**	1 2 3 4 5	1 2 3 4 5
**30. Acting decisively in a crisis**	1 2 3 4 5	1 2 3 4 5
**31. Delegating appropriately**	1 2 3 4 5	1 2 3 4 5
**32. Being receptive to others in the team**	1 2 3 4 5	1 2 3 4 5
**33. Willingness to listen/take advice**	1 2 3 4 5	1 2 3 4 5
**34. Alertness/ being 'on the ball'**	1 2 3 4 5	1 2 3 4 5

**35. Managing risk appropriately**	1 2 3 4 5	1 2 3 4 5
**36. Organisational skills/efficiency**	1 2 3 4 5	1 2 3 4 5
**37. Checking and re-checking in error-prone situations**	1 2 3 4 5	1 2 3 4 5
**38. Being available & perceived as available**	1 2 3 4 5	1 2 3 4 5
**39. Giving constructive feedback**	1 2 3 4 5	1 2 3 4 5
**40. Allowing others to take over where appropriate**	1 2 3 4 5	1 2 3 4 5
**41. Being able to minimise distractions in the clinical environment**	1 2 3 4 5	1 2 3 4 5
**42. Being able to follow instructions where appropriate**	1 2 3 4 5	1 2 3 4 5
**43. Having a sense of 'togetherness' within the team**	1 2 3 4 5	1 2 3 4 5
**44. Being thorough/paying attention to detail**	1 2 3 4 5	1 2 3 4 5
**45. Gathering information adequately and competently**	1 2 3 4 5	1 2 3 4 5
**46. Conscientiousness**	1 2 3 4 5	1 2 3 4 5
**47. Prioritisation/multi tasking**	1 2 3 4 5	1 2 3 4 5
**48. Acting appropriately if hazards are noticed**	1 2 3 4 5	1 2 3 4 5
**49. Contingency planning with clearly defined levels of care (safety-netting)**	1 2 3 4 5	1 2 3 4 5
**50. Regularly re-reviewing the situation**	1 2 3 4 5	1 2 3 4 5
**51. Having & attending frequent/regular team meetings**	1 2 3 4 5	1 2 3 4 5
**52. Caring about the patient**	1 2 3 4 5	1 2 3 4 5
**53. Empathy**	1 2 3 4 5	1 2 3 4 5
**54. Not becoming frustrated with patients one cannot solve**	1 2 3 4 5	1 2 3 4 5
**55. Not thinking of re-attenders as a nuisance**	1 2 3 4 5	1 2 3 4 5
**56. Thinking "*how am I today?*" (tiredness, stress, etc)**	1 2 3 4 5	1 2 3 4 5

57. Being confident in decision-making	1 2 3 4 5	1 2 3 4 5
58. Changing one's behaviour in response to tiredness	1 2 3 4 5	1 2 3 4 5
59. Being aware of unsafe members in the team	1 2 3 4 5	1 2 3 4 5
60. Being aware of others around you	1 2 3 4 5	1 2 3 4 5
61. Recognising a sick patient	1 2 3 4 5	1 2 3 4 5
62. Having appropriate diagnostic skills	1 2 3 4 5	1 2 3 4 5
63. Managing overconfident/blasé juniors	1 2 3 4 5	1 2 3 4 5
64. Learning from previous mistakes	1 2 3 4 5	1 2 3 4 5
65. Team back-up behaviours (supporting others)	1 2 3 4 5	1 2 3 4 5
66. Preparedness	1 2 3 4 5	1 2 3 4 5

Finally, please state if there are any other safety skills that you feel are missing from this list.

For each skill you mention, please also rate its importance/relevance to being a safe GP and its trainability

OTHER SAFETY SKILLS NOT MENTIONED ABOVE (continue on separate page if necessary)

1.

2.

3.

4.

5.
